# PIK3CA mutations enhance the adipogenesis of ADSCs in facial infiltrating lipomatosis through TRPV1

**DOI:** 10.1016/j.isci.2024.110467

**Published:** 2024-07-05

**Authors:** Hongrui Chen, Bin Sun, Wei Gao, Yajing Qiu, Wei Wei, Yongguo Li, Wei Ye, Haoliang Song, Chen Hua, Xiaoxi Lin

**Affiliations:** 1 Department of Plastic & Reconstructive Surgery, Shanghai Ninth People’s Hospital, Shanghai Jiao Tong University School of Medicine, Shanghai, P.R. China; 2 Shanghai Jiatan Pharmatech Co, LTD, Shanghai, China

**Keywords:** cell biology, molecular biology, physiology, stem cells research

## Abstract

Facial infiltrating lipomatosis (FIL) is a congenital disorder. The pathogenesis of FIL is associated with *PIK3CA* mutations, but the underlying mechanisms remain undetermined. We found that the adipose tissue in FIL demonstrated adipocytes hypertrophy and increased lipid accumulation. All adipose-derived mesenchymal stem cells from FIL (FIL-ADSCs) harbored *PIK3CA* mutations. Moreover, FIL-ADSCs exhibited a greater capacity for adipogenesis. Knockdown of *PIK3CA* resulted in a reduction in the adipogenic potential of FIL-ADSCs. Furthermore, WX390, a dual-target PI3K/mTOR inhibitor, was found to impede *PIK3CA*-mediated adipogenesis both *in vivo* and *in vitro*. RNA sequencing (RNA-seq) revealed that the expression of transient receptor potential vanilloid subtype 1 (TRPV1) was upregulated after PI3K pathway inhibition, and overexpression or activation of TRPV1 both inhibited adipogenesis. Our study showed that *PIK3CA* mutations promoted adipogenesis in FIL-ADSCs and this effect was achieved by suppressing TPRV1. Pathogenesis experiments suggested that WX390 may serve as an agent for the treatment of FIL.

## Highlights

• FIL-ADSCs demonstrated an enhanced adipogenesis compared to CON-ADSCs
• *PIK3CA* mutations enhanced FIL-ADSCs adipogenesis via TRPV1 suppression
• WX390, a PI3K/mTOR inhibitor, notably inhibited adipogenesis
• Activation or overexpression of TRPV1 impaired the adipogenesis of FIL-ADSCs

## Graphical abstract

**Figure:**
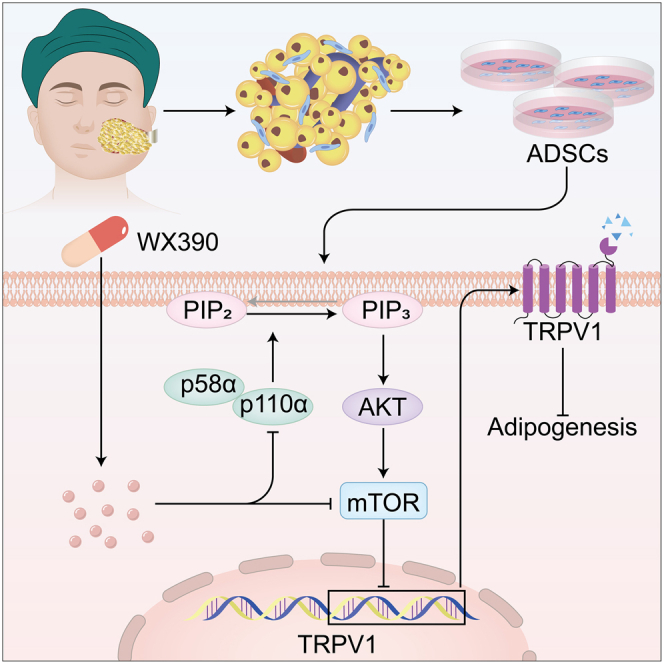

## Introduction

Facial infiltrating lipomatosis (FIL) is a rare disorder characterized by congenital unilateral facial enlargement.^1^ It is a nonhereditary condition with no apparent gender predilection. Although facial asymmetry is often present at birth, abnormal unilateral overgrowth is most noticeable during infancy or adolescence. Hemifacial adipose tissue hyperplasia is frequently present.^2^ Other oral problems, such as mucosal neuromas, macrodontia, and abnormal tooth morphology, are also common.^3^^,^^4^ Histologically, mature unencapsulated adipocytes grow invasively and diffusely infiltrate the surrounding structures, including the cheeks, parotid glands, tongues, masticatory muscles, and lips.^5^ FIL not only significantly compromises facial aesthetics, but it may also impair crucial facial functions including swallowing, breathing, chewing, and vision. In severe cases, it can even pose a threat to the patient’s life.^6^ Although surgery is currently the primary strategy for the treatment of FIL, the infiltrative growth of adipose tissue limits surgical outcomes. Incomplete resection of lipomatosis is often accompanied by a high recurrence rate, yet complete removal of focal adipose tissue is difficult to achieve because it may cause dramatic damage to the muscles, facial nerve, and glands.^1^^,^^5^^,^^7^ Thus, effective therapies based on pathophysiological mechanisms are urgently needed.

Uncovering the mechanisms of excessive adipose tissue accumulation in FIL will facilitate the development of therapeutic strategies. Adipogenesis is a process in which pluripotent stem cells differentiate into mature adipocytes,^8^ and abnormalities in this process are associated with a range of human diseases, such as obesity, fatty liver, and lipoma formation.^9^^,^^10^^,^^11^ This process is regulated by multiple signal transduction pathways, one of which is the PI3K-AKT pathway.^12^ Phosphoinositide-3 kinases (PI3K) are essential lipid kinases that modulate downstream pathways associated with critical cellular activities such as proliferation, metabolism, and differentiation.^13^ Several studies have revealed that the PI3K-AKT pathway is involved in adipogenesis in adipocyte precursor cells and adipose-derived mesenchymal stem cells (ADSCs).^14^^,^^15^

Class IA PI3Ks include the p110α catalytic subunit and the p85α regulatory subunit. The catalytic subunit p110α, which is encoded by phosphatidylinositol 3-kinase catalytic subunit alpha (*PIK3CA*), can catalyze the phosphorylation of PtdIns-4,5-P_2_ (PIP_2_) to generate PtdIns-3,4,5-P_3_ (PIP_3_). This phospholipid can act as a second messenger and further interact with downstream signaling proteins.^16^ Normally, p85α binds to p110α and inhibits its catalytic activity. *PIK3CA* gain-of-function mutations compromise the ability of p85α to inhibit p110α, resulting in enhanced PI3K signaling and a variety of overgrowth disorders.^17^^,^^18^ Recently, postzygotic *PIK3CA* mutations have been detected in adipose tissue from FIL patients.^19^^,^^20^^,^^21^ However, the precise molecular mechanisms linking *PIK3CA* mutations and the excessive adipogenesis in FIL are not yet entirely understood.

In this study, we demonstrated that *PIK3CA* activating mutations promoted adipogenesis in ADSCs isolated from FIL patients (FIL-ADSCs), and this effect was achieved via PI3K/AKT/TRPV1 pathway. This work revealed a potential mechanism for the development of lipomatosis and showed that the PI3K/mTOR dual-target inhibitor WX390 is a promising therapeutic option for treating FIL.

## Results

### The adipose tissue of FIL patients exhibited increased lipid accumulation and enhanced activation of the PI3K pathway

**Figure 1:**
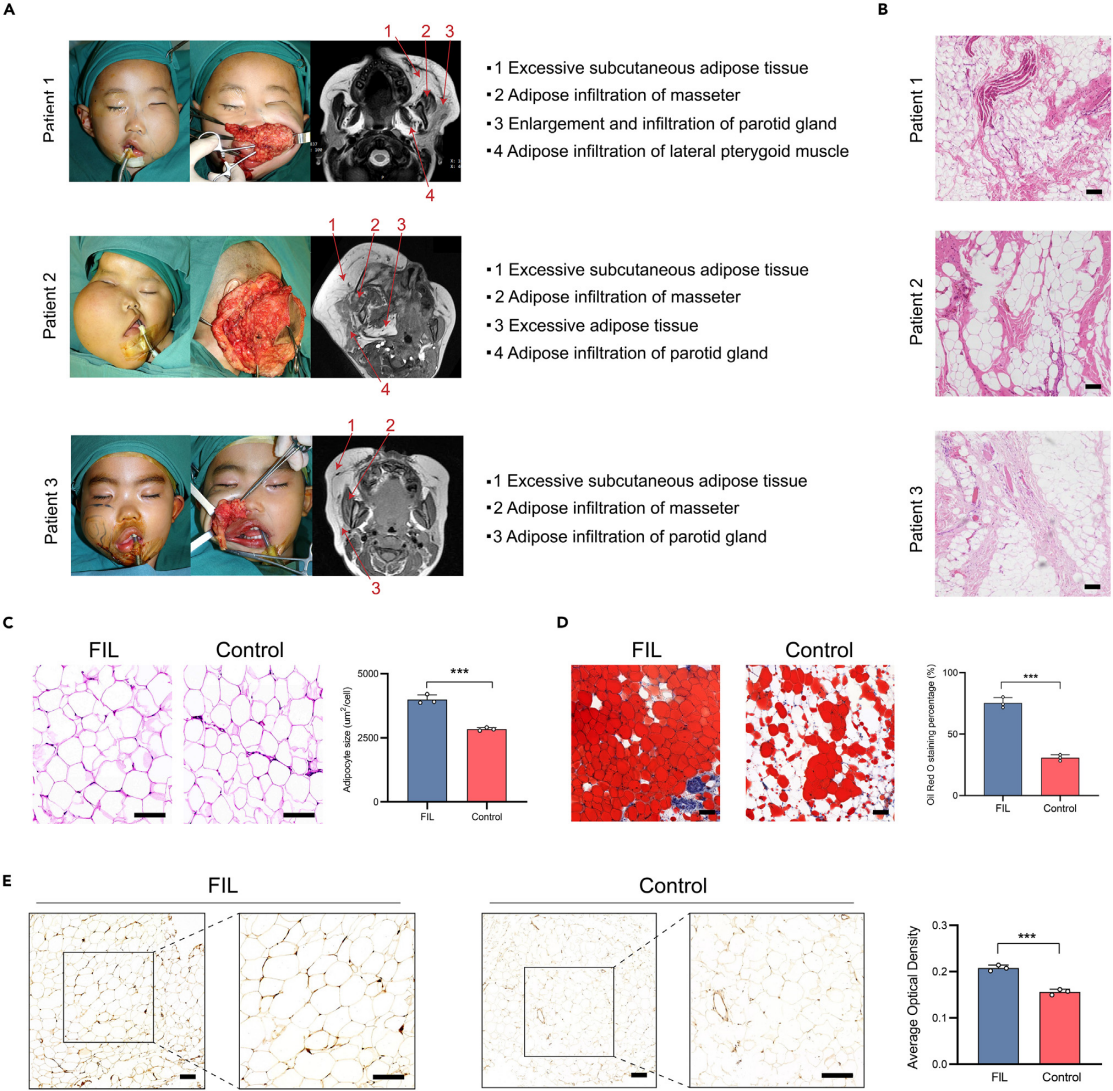
Histological features of adipose tissue of FIL patients and control (A) Intraoperative photographs of the three FIL patients and affected facial regions assessed with magnetic resonance imaging. (B) Representative H&E staining of adipose tissue of FIL patients. (C) Comparison of adipocytes size of FIL patients and control. (D) Oil red O staining and quantitative analysis of the proportion of the oil red O staining area within the whole visual field of adipose tissue from the FIL and control groups (representing the area of the lipid droplets). (E) IHC staining for *p*-AKT in adipose tissue from the FIL and control groups and the protein expression level analysis. Data are shown as the mean ± SD. ∗∗∗*p* < 0.001. Scale bar: 200 μm.

We identified three pediatric patients with FIL who exhibited abundant subcutaneous fat accumulation that was visible intraoperatively. All patients had magnetic resonance imaging demonstrating fat infiltration into the surrounding muscles and glands, with local tissue enlargement and anatomical abnormalities (Figure 1A). Histologically, surgical resection specimens from all patients showed diffuse infiltration of mature, non-encapsulated fat cells into the surrounding soft tissue (Figure 1B). To further compare the morphological differences between the adipose tissue in FIL and normal facial adipose tissue, we chose facial adipose tissue from patients undergoing surgery due to skin lesions as a control for further observation. We performed hematoxylin and eosin (H&E) staining and oil red O staining on both. H&E staining showed that adipocytes in the FIL tissue were hypertrophic compared to controls (Figure 1C). Oil red O staining showed increased lipid accumulation in the FIL tissue (Figure 1D). Additionally. Immunohistochemistry staining (IHC) showed that FIL tissue had high expression of *p*-AKT (Figure 1E), indicating enhanced activity of the PI3K-AKT pathway.

### FIL-ADSCs harbored PIK3CA mutations

**Figure 2:**
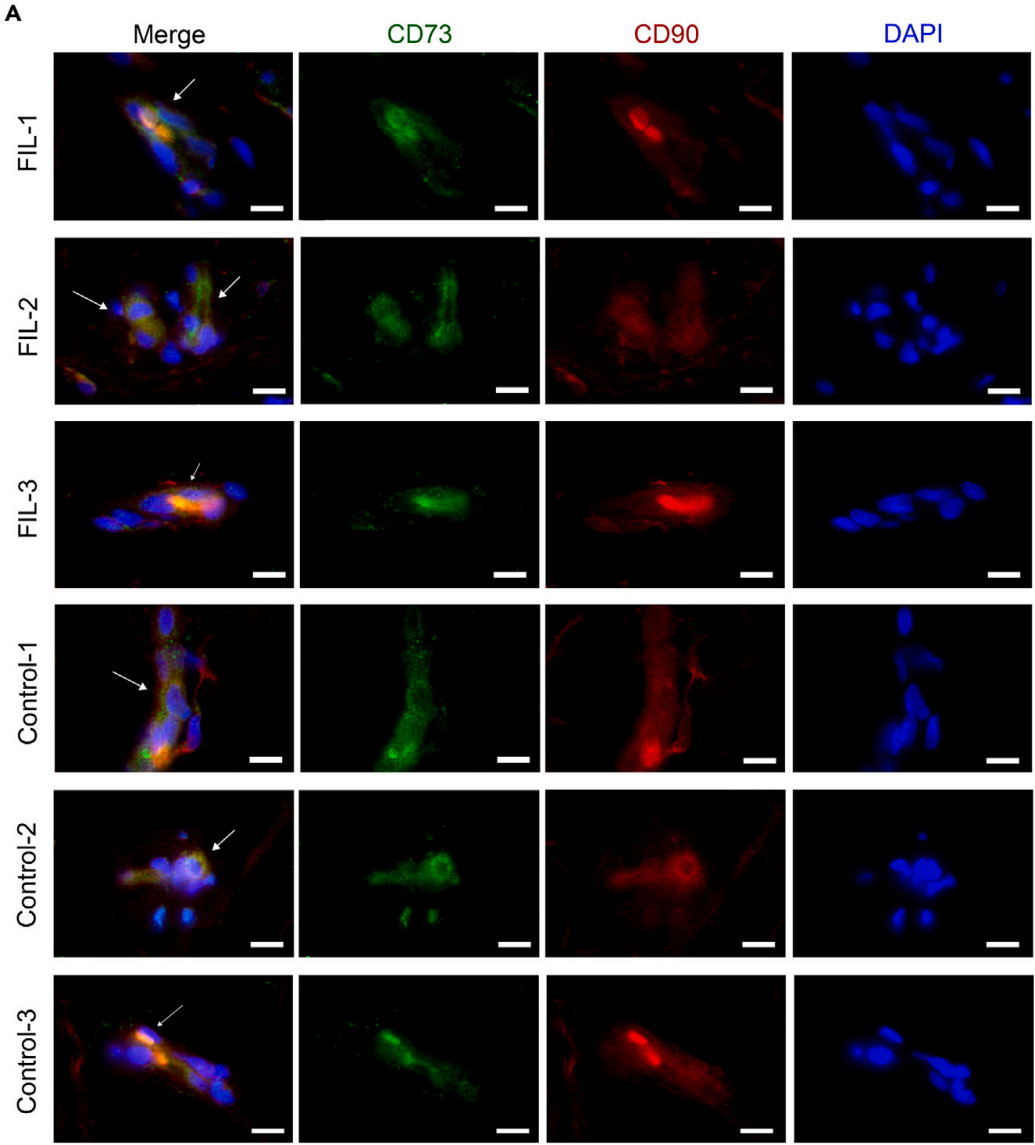
Immunofluorescence staining showing the presence of the ADSCs in the subcutaneous adipose tissues of FIL patients and control (A) Staining results in adipose tissue sections of three FIL patients and three control patients. Scale bar: 10 μm.

**Figure 3:**
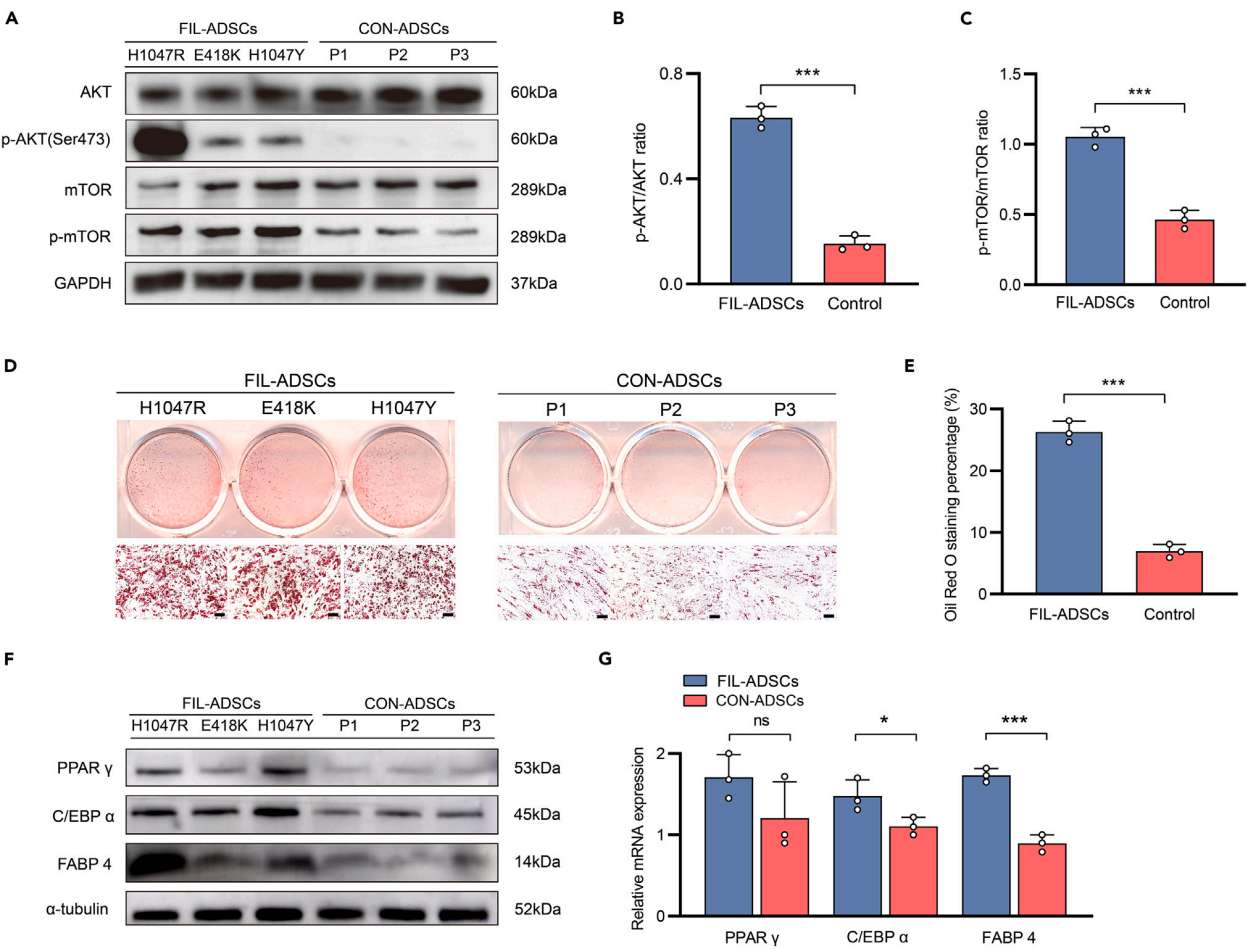
FIL-ADSCs with different PIK3CA mutations had a stronger adipogenic capacity (A) Western blotting analysis of proteins downstream of the PI3K-AKT pathway in FIL-ADSCs and CON-ADSCs. GAPDH protein levels were used as a reference standard. (B and C) Analysis of band density. Protein levels were quantified using ImageJ software and represented by a histogram (fold change of *p*-AKT/AKT and *p*-mTOR/mTOR). (D) Oil red O staining of FIL-ADSCs and CON-ADSCs after adipogenic induction. Scale bar: 100 μm. (E) Quantitative analysis of the proportion of the oil red O staining area. (F) Western blot analysis of PPAR γ, C/EBP α, and FABP 4 in FIL-ADSCs and CON-ADSCs (3 strains of FIL-ADSCs vs. 3 strains of CON-ADSCs) after the administration of adipogenic differentiation solution A for 1 day α-Tubulin protein levels were used as a reference standard. (G) Detection of PPAR γ, C/EBP α, and FABP 4 in FIL-ADSCs and CON-ADSCs (3 strains of FIL-ADSCs vs. 3 strains of CON-ADSCs) by quantitative real-time PCR (RT-qPCR) after adipogenic induction for 1 day. GAPDH mRNA served as a reference standard. ∗*p* < 0.05, ∗∗∗*p* < 0.001. Experiments were independently replicated at least three times with similar results (biological replicates). Data were analyzed by unpaired two-sided Student’s t tests (B, C, E, and G) and are presented as mean ± SD with three replicate experiments.

We first confirmed the presence of ADSCs in adipose tissue through immunofluorescence (Figure 2A). Considering the impact of both anatomical sites and age on the functional status of ADSCs,^22^ we used ADSCs isolated from normal facial subcutaneous fat of other pediatric patients with similar age (See Table S1) who underwent excision of facial skin lesions as control (CON-ADSCs). Both FIL-ADSCs and CON-ADSCs displayed a similar spindle-shaped fibroblast-like morphology (See Figure S1A) and expressed typical stem cell markers^23^ (See Figure S1B). Whole-exome sequencing revealed three FIL-ADSC strains carrying *PIK3CA H1047R*, *E418K*, and *H1047Y* mutations, respectively. No mutations were found in CON-ADSCs. Western blotting demonstrated increased levels of phosphorylated AKT and mTOR in FIL-ADSCs compared to CON-ADSCs, indicating excessive activation of the PI3K-AKT pathway (Figures 3A–3C). Furthermore, FIL-ADSCs showed enhanced proliferative capacity (See Figure S1C).

### FIL-ADSCs demonstrated a high capacity for adipogenic differentiation

To further investigate whether excess lipid formation in lipomatosis was associated with FIL-ADSCs, we compared the adipogenic capacity of FIL-ADSCs and CON-ADSCs. Following 12 days of adipogenic differentiation induction, oil red O staining revealed that FIL-ADSCs exhibited an increased lipid droplet formation than the control (Figures 3D and 3E). Peroxisome proliferator-activated receptor gamma (PPAR γ), CCAAT enhancer binding protein alpha (C/EBP α), and fatty acid-binding protein 4 (FABP 4) are three crucial transcriptional regulators involved in the regulation of the adipogenic differentiation process.^24^^,^^25^ Consistently, we observed higher expression levels of PPAR γ, C/EBP α, and FABP 4 in FIL-ADSCs than in CON-ADSCs at both the mRNA and protein levels (Figures 3F and 3G). These findings suggested that the FIL-ADSCs had enhanced adipogenic capacity than the CON-ADSCs.

### PIK3CA was critical for the aberrant adipogenesis of FIL-ADSCs

**Figure 4:**
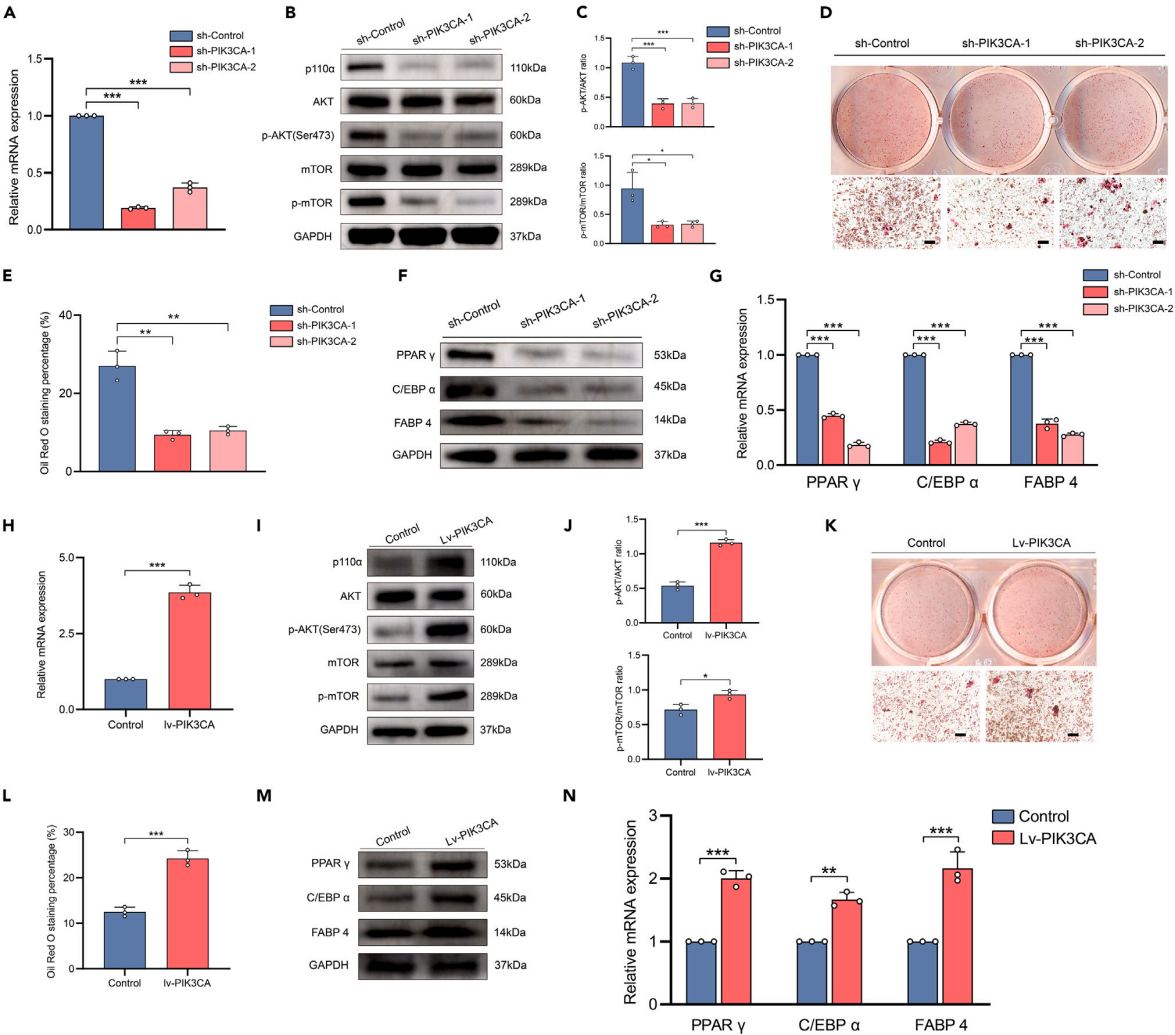
PIK3CA was crucial for the adipogenesis of ADSCs (A) RT-qPCR results showed the silencing of PIK3CA after lentivirus transfection. GAPDH mRNA served as a reference standard. (B and C) Western blot analysis of proteins downstream of the PI3K-AKT pathway in PIK3CA-knockdown FIL-ADSCs. GAPDH protein levels were used as a reference standard. (D) Oil red O staining showed reduced lipid synthesis in PIK3CA-knockdown FIL-ADSCs. (E) Quantitative assessment of oil red O staining area. (F and G) Gene expression analysis examining PPAR γ, C/EBP α, and FABP 4 levels using western blotting and RT-qPCR in FIL-ADSCs and PIK3CA-knockdown FIL-ADSCs after adipogenic induction for one day. GAPDH mRNA served as a reference standard. (H) RT-qPCR results showed the overexpression of PIK3CA after lentivirus transfection. GAPDH mRNA served as a reference standard. (I and J) Western blot analysis of proteins downstream of the PI3K-AKT pathway in PIK3CA-overexpression CON-ADSCs. GAPDH protein levels were used as a reference standard. (K) Oil red O staining showed increased lipid synthesis in PIK3CA-overexpression CON-ADSCs. (L) Quantitative assessment of oil red O staining area. (M and N) Gene expression analysis examining PPAR γ, C/EBP α, and FABP 4 levels using western blotting and RT-qPCR in CON-ADSCs and PIK3CA-overexpression CON-ADSCs after adipogenic induction for one day. GAPDH mRNA served as a reference standard. ∗*p* < 0.05, ∗∗*p* < 0.01,∗∗∗*p* < 0.001. Scale bar: 100 μm. Experiments were independently replicated at least three times with similar results (biological replicates). Data were analyzed by unpaired two-sided Student’s t tests (H, J, L, and N) or one-way ANOVA (A, C, E, and G), and are presented as mean ± SD with three replicate experiments.

The PI3K-AKT pathway is a crucial signaling pathway regulating adipogenesis.^26^ Therefore, we explored whether the greater adipogenic ability in FIL-ADSCs was attributable to PIK3CA-activating mutations. We first knocked down PIK3CA via lentivirus-mediated shRNA transfection in FIL-ADSCs. Quantitative real-time PCR (RT-qPCR) analysis revealed a significant decrease in the expression of PIK3CA after viral transduction (Figure 4A), and western blotting demonstrated downregulation of p110α protein expression and decreased phosphorylation levels of the downstream signaling proteins of PIK3CA (Figures 4B and 4C). Following 12 days of induction, oil red O staining revealed that knockdown of PIK3CA impaired the lipogenic ability of FIL-ADSCs (Figures 4D and 4E), and the expression levels of PPAR γ, C/EBP α, and FABP 4 were also reduced after PIK3CA knockdown (Figures 4F and 4G). We further overexpressed PIK3CA in CON-ADSCs to mimic the effect of PIK3CA mutations (overactivation of downstream pathways). We used RT-qPCR and western blotting assay to confirm overexpression efficiency (Figures 4H–4J), and subsequently performed adipogenic induction. After 12 days, oil red O staining showed that CON-ADSCs overexpressing PIK3CA had more lipid accumulation (Figures 4K and 4L). Consistently, the expression levels of PPAR γ, C/EBP α, and FABP 4 were higher in the group overexpressing PIK3CA (Figures 4M and 4N). These results indicated that PIK3CA played a crucial role in the adipogenic differentiation of ADSCs.

### The PI3K/mTOR dual-target inhibitor WX390 diminished the adipogenic capacity of FIL-ADSCs

**Figure 5:**
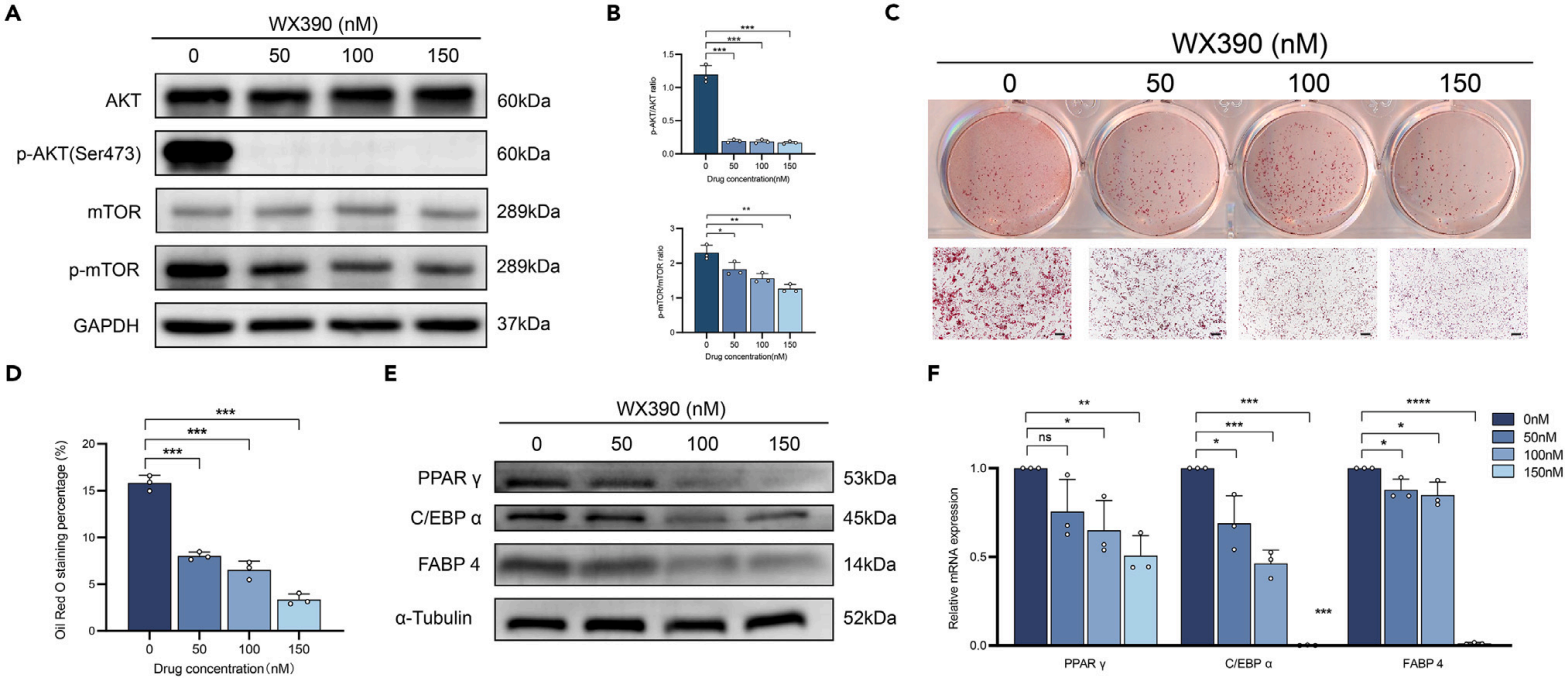
WX390 inhibited the adipogenic differentiation of FIL-ADSCs (A and B) The phosphorylation levels of AKT and mTOR were determined by western blotting in FIL-ADSCs after WX390 treatment for 8 h. (C) Oil red O staining of FIL-ADSCs after the administration of WX390. (D) Quantitative assessment of oil red O staining area. (E and F) The expression of PPAR γ, C/EBP α, and FABP 4 was determined by western blotting and RT‒qPCR. ∗*p* < 0.05, ∗∗*p* < 0.01, ∗∗∗*p* < 0.001. Scale bar: 100 μm. Experiments were independently replicated at least three times with similar results (biological replicates). Data were analyzed by one-way ANOVA (B, D, and F), and are presented as mean ± SD with three replicate experiments.

Previous research has demonstrated that the PI3Kα-targeted inhibitor BYL719 suppresses adipose tissue overgrowth.^13^ WX390 is a dual-target PI3K/mTOR inhibitor with over 30-fold higher *in vitro* PI3K enzyme activity than BYL719,^27^ and it inhibits PI3Kα inhibitor-induced resistance through moderate inhibition of mTOR. To investigate whether inhibition of p110α and mTOR impedes aberrant adipogenesis, we treated FIL-ADSCs with WX390. We observed that WX390 effectively suppressed the phosphorylation levels of AKT and mTOR in a dose-dependent manner (Figures 5A and 5B). Furthermore, WX390 significantly suppressed lipid synthesis in FIL-ADSCs upon adipogenic induction (Figures 5C and 5D). Consistently, a dose-dependent decrease in the expression of PPARγ, C/EBPα, and FABP4 was also observed after exposure to WX390 (Figures 5E and 5F).

### WX390 suppressed adipogenesis in a murine model

**Figure 6:**
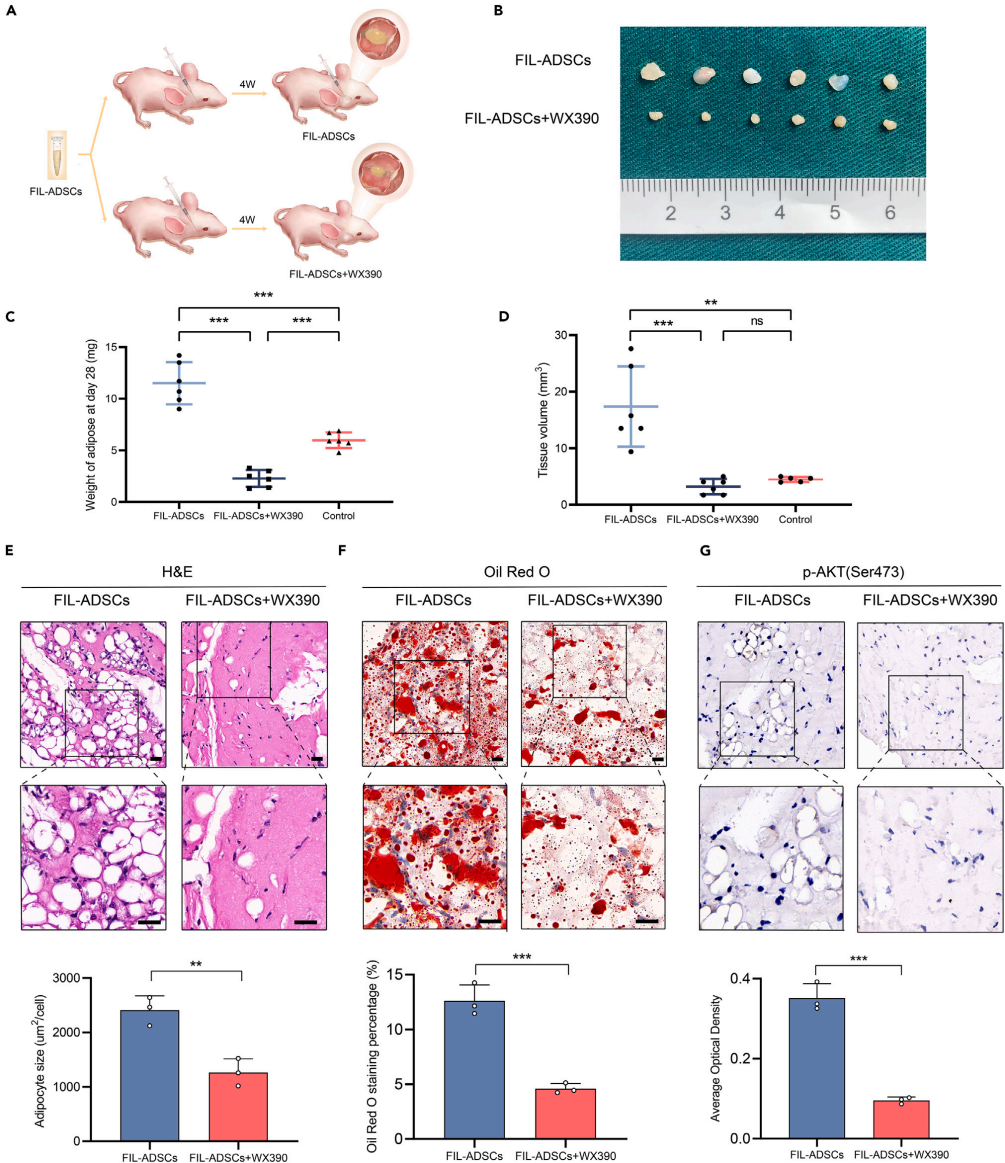
WX390 retained its inhibitory effect on adipogenesis *in vivo* (A) Schematic depiction of the experimental process for the murine model. (B) The macroscopic appearance of Matrigel implants harvested from mice treated with intragastric administration of saline or WX390 (0.1 mg/kg body weight) every day from the day after subcutaneous implantation. The Matrigel implants were collected on day 28. (C and D) Statistical analysis of implant volume and weight. (E) H&E staining of Matrigel implants collected on day 28 and quantification of adipocytes size. (F) Oil red O staining of Matrigel implants from the WX390 treatment group and control group and quantification of oil red O staining area. (G) Immunohistochemical staining for *p*-AKT of Matrigel implants from the WX390 treatment group and control group and protein expression level analysis. ∗∗*p* < 0.01, ∗∗∗*p* < 0.001. Scale bar: 100 μm. Data were analyzed by unpaired two-sided Student’s t tests (E, F, and G) or one-way ANOVA (C and D), and are presented as mean ± SD with three replicate experiments.

To further investigate the *in vivo* effects of WX390, we established a murine xenograft model by injecting Matrigel containing FIL-ADSCs and CON-ADSCs into the facial region of immunodeficient female mice (Figure 6A). The mice injected with FIL-ADSCs were randomly divided into a medicated group (*n* = 6) and a null group (*n* = 6). Mice in the medicated group were given 150 μL of WX390 working solution daily by gavage and the mice in the null group were given 150 μL of saline. The concentration of WX390 in the working solution was 0.01 mg/mL. We observed that after 4 weeks, the volume and weight of implants in the WX390 treatment group were significantly lower than that in the untreated group (Figures 6B–6D and S2A). Moreover, the mice tolerated the WX390 treatment well, with no deaths and no apparent damage to liver or kidney morphology (See Figures S2B and S2C). The body weight of WX390-treated mice was not different from that of the mice in the other groups (See Figure S2D). H&E and oil red O staining revealed a dramatic decrease in adipocytes size and lipid accumulation in the implant treated with WX390 (Figures 6E and 6F). Besides, IHC showed that *p*-AKT level was significantly lower in WX390 treated implants than null group (Figure 6G).

### TRPV1 expression was regulated by PI3K-AKT in FIL-ADSCs

**Figure 7:**
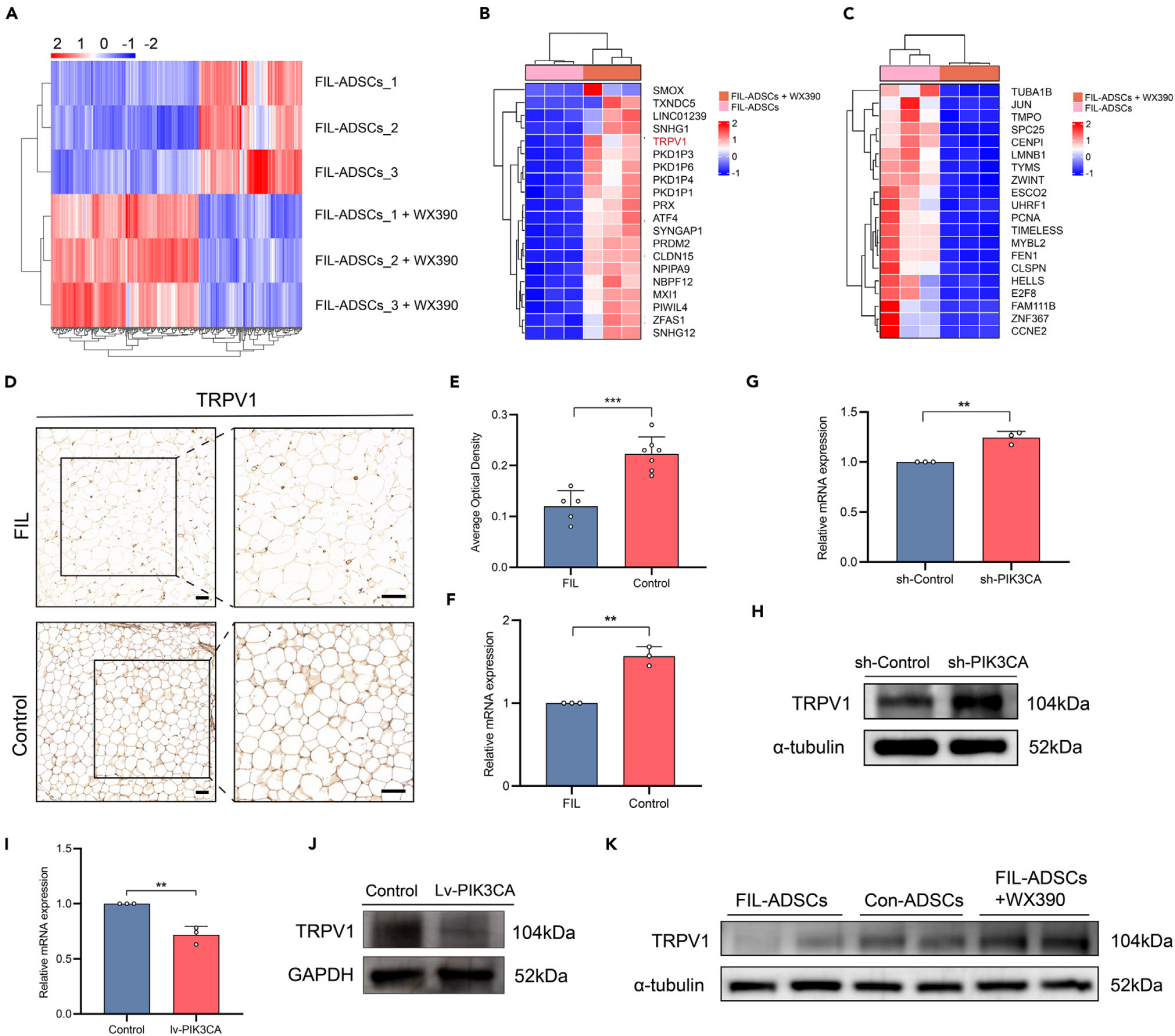
TRPV1 was regulated by PI3K-AKT pathway (A) Heatmap showing the transcriptional expression profiles of FIL-ADSCs and FIL-ADSCs + WX390. (B) Heatmap of the top 20 genes significantly upregulated following WX390-treated. (C) Heatmap of the top 20 genes significantly downregulated following WX390-treated. (D) Immunohistochemical staining for TRPV1 of adipose tissue from FIL patients and control patients. (E) The optical density was quantified by ImageJ. The TRPV1 expression level in adipose tissue of FIL (*n* = 5) and control (*n* = 7). (F) The mRNA level of TRPV1 in adipose tissue of FIL and control. (G and H) The mRNA and protein levels of TRPV1 in FIL-ADSCs subjected to PIK3CA knockdown were determined by RT-qPCR and western blotting. (I and J) The mRNA and protein levels of TRPV1 in CON-ADSCs subjected to PIK3CA overexpression were determined by RT‒qPCR and western blotting. (K) Protein levels of TRPV1 in FIL-ADSCs, CON-ADSCs, and FIL-ADSCs+WX390 (24 h, 150 nM). ∗∗*p* < 0.01, ∗∗∗*p* < 0.001. Scale bar: 200 μm. Experiments were independently replicated at least three times with similar results (biological replicates). Data were analyzed by unpaired two-sided Student’s t tests (E, G, F, and I) and are presented as mean ± SD with three replicate experiments.

The PI3K-AKT pathway represents a critical role in cellular signaling networks, regulating the expression of numerous downstream effectors.^28^^,^^29^ To further investigate which effector molecules PIK3CA mutations act on via the PI3K-AKT pathway to regulate adipogenesis, we performed comparative RNA sequencing (RNA-seq) of FIL-ADSCs (*n* = 3) and WX390-treated FIL-ADSCs (*n* = 3). Overall, treatment with WX390 resulted in the upregulation of 152 genes and the downregulation of 217 genes (Figure 7A). Of note, we observed significant upregulation of transient receptor potential vanilloid subtype 1 (TRPV1) following WX390 treatment (Figures 7B and 7C). TRPV1 is a nonselective cation channel with high calcium permeability that can be activated by capsaicin.^30^ Previous studies have discovered that activation of this channel can counteract adipogenesis and obesity,^31^^,^^32^^,^^33^ and other research has demonstrated that activation of the PI3K-AKT pathway can regulate TRPV1 expression.^34^^,^^35^^,^^36^ IHC of 5 adipose tissues from FIL and 7 normal facial adipose tissues showed significantly lower levels of global TRPV1 expression in FIL than in normal facial adipose tissues (Figures 7D and 7E). TRPV1 mRNA level was also lower in FIL than normal facial adipose tissues (Figure 7F).

To further validate whether TRPV1 expression in ADSCs was regulated by the PI3K pathway, we analyzed the expression of TRPV1 in different conditions. Knockdown of PIK3CA resulted in increased TRPV1 expression at both the mRNA and protein levels (Figures 7G and 7H), whereas overexpression of PIK3CA inhibited the expression of TRPV1 (Figures 7I and 7J). Moreover, TRPV1 was significantly increased following treatment with WX390 (Figure 7K). These findings suggested that activation of the PI3K pathway may suppress TRPV1 expression, while inhibition of this pathway abolished this effect.

### Overexpression or activation of TRPV1 decreased the adipogenic capacity of FIL-ADSCs

**Figure 8:**
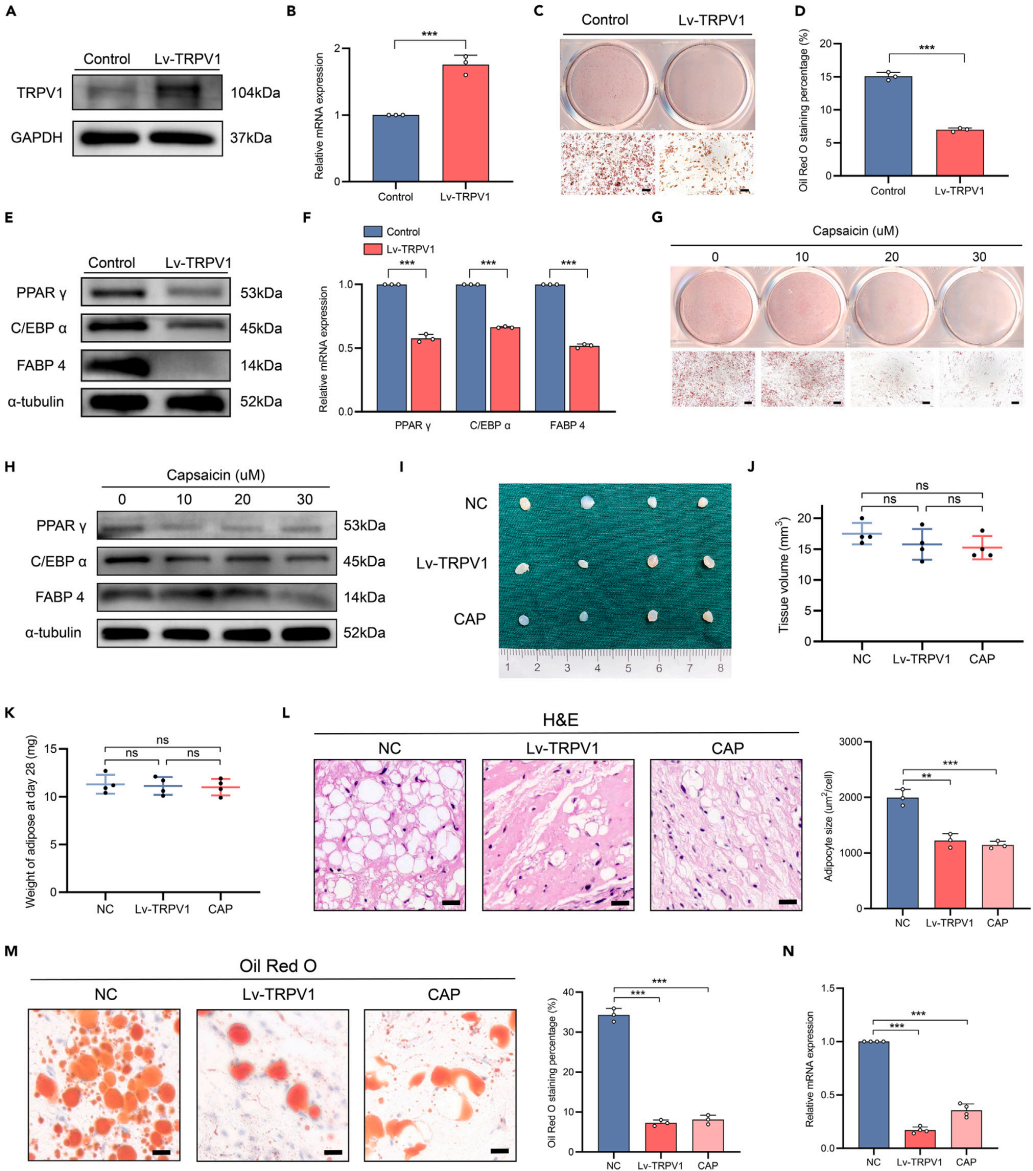
TRPV1 inhibited adipogenesis of FIL-ADSCs (A and B) RT-qPCR and western blotting verified the overexpression of TRPV1 after transfection of the lentivirus into FIL-ADSCs with PIK3CA mutations. (C and D) Oil red O staining showed that overexpression of TRPV1 impaired adipogenesis of FIL-ADSCs. (E and F) The mRNA and protein levels of PPAR γ, C/EBP α, and FABP 4 in FIL-ADSCs overexpressing TRPV1 were determined by RT-qPCR and western blotting. (G) Oil red O staining of FIL-ADSCs treated with capsaicin. (H) Western blotting analysis of PPAR γ, C/EBP α, and FABP 4 in capsaicin-treated FIL-ADSCs. α-tubulin protein levels were used as a reference standard. (I) The macroscopic appearance of Matrigel implants from NC, Lv-TRPV1 and CAP group. (J) Volume of xenograft of NC, Lv-TRPV1, and CAP group. (K) Weight of xenograft of NC, Lv-TRPV1, and CAP group. (L) H&E staining of Matrigel implants and quantification of adipocytes size. (M) Oil red O staining of Matrigel implants and quantification of oil red O staining area. (N) The mRNA expression levels of PPAR γ of xenograft were determined by RT-qPCR. ∗∗∗*p* < 0.001. Scale bar: 100 μm. Experiments were independently replicated at least three times with similar results (biological replicates). Data were analyzed by unpaired two-sided Student’s t tests (B, D, and F) or one-way ANOVA (J, K, L, M, and N), and are presented as mean ± SD with three replicate experiments.

Previous studies have elucidated the involvement of TRPV1 in regulating transcription factors associated with adipogenesis and combating obesity.^37^^,^^38^^,^^39^ To investigate whether TRPV1 could modulate the adipogenic capacity of FIL-ADSCs, we transfected a TRPV1 overexpression lentivirus (Lv-TRPV1) into FIL-ADSCs to induce TRPV1 overexpression (Figures 8A and 8B). We found that FIL-ADSCs overexpressing TRPV1 exhibited significantly reduced adipogenic capacity during the same induction period (Figures 8C and 8D). Consistently, the adipogenic markers PPAR γ, FABP 4, and C/EBP α were downregulated at both the mRNA and protein levels (Figures 8E and 8F). Capsaicin, the most important ingredient in chili peppers, is an exogenous agonist of TRPV1.^32^ To investigate the effect of TRPV1 activation on adipogenesis, we treated FIL-ADSCs with capsaicin.^37^ A reduction in lipid synthesis and a dose-dependent decrease in the expression of adipogenic markers in capsaicin-treated FIL-ADSCs were observed (Figures 8G and 8H).

To determine whether TRPV1 inhibited adipogenesis *in vivo*, we once again injected Matrigel containing FIL-ADSCs subcutaneously into the facial area of mice. The xenografts were divided into three groups: the group with FIL-ADSCs transfected with empty lentivirus (NC), the group with FIL-ADSCs overexpressing TRPV1 (Lv-TRPV1), and the group fed with 0.01% capsaicin in diet after injection of FIL-ADSCs transfected with empty virus (CAP).^33^ After four weeks, we euthanized the mice and collected the implants. There were no significant differences in volume and weight among the three groups of implants (Figures 8I–8K). The FIL-ADSCs/Matrigel implants were then sectioned and stained. H&E staining revealed that all implants in the NC group had a large number of adipocytes, whereas only a minimal number of adipocytes were found in the implants of the Lv-TRPV1 group and the CAP group (Figure 8L). Oil red O staining showed that the staining area of the NC group implants was significantly higher than that of the Lv-TRPV1 group and the CAP group, indicating more lipid accumulation (Figure 8M). The mRNA expression levels of adipogenic markers PPAR γ were also decreased in the Lv-TRPV1 and CAP groups (Figure 8N). Overall, these results suggested that the activation or overexpression of TRPV1 inhibited the adipogenic differentiation of FIL-ADSCs, while PIK3CA mutations abolished this effect.

## Discussion

FIL is a rare condition that presents with various clinical features and poses a significant challenge in terms of management due to its unpredictable nature. Surgical intervention remains the primary treatment option, with subtotal resection of hypertrophied soft tissue and bone being the recommended approach.^1^^,^^40^^,^^41^ However, complete resection is often not feasible due to extensive infiltration of adipose tissue and proximity to vital structures. Local recurrence is common and can be difficult to manage.^2^^,^^7^^,^^42^ Therefore, it is imperative to explore alternative treatment strategies beyond surgery. While previous studies on FIL have focused on clinical phenotypes and genotypes, there has been no exploration of molecular pathogenesis. Our study provides evidence that PIK3CA mutations promote the adipogenic capacity of ADSCs in FIL through downregulation of TRPV1.

PIK3CA-activating mutations have been found to be associated with a variety of overgrowth disorders, collectively known as the PIK3CA-related overgrowth spectrum (PROS).^43^ These disorders often present with skeletal deformities, vascular malformations, and nerve enlargement.^44^ Adipose tissue overgrowth, including fibroadipose hyperplasia, hemihyperplasia multiple lipomatosis, fibroadipose vascular anomaly, and FIL, are also important components of PROS.^45^ We detected three different PIK3CA mutations in ADSCs isolated from lipomatosis tissue from three patients, further suggesting that similar phenotypes may be caused by multiple genotypes, which is a feature of PROS.^46^ All three FIL-ADSC strains exhibited hyperactivation of the PI3K-AKT pathway compared to the control group. Among the observed mutations, H1047R was considered the hotspot mutation, with strong oncogenic capacity,^47^ and FIL-ADSCs carrying this mutation also had the highest phosphorylation levels of downstream substrates. Previous studies have shown that the PI3K-AKT pathway is critical to adipogenesis,^48^^,^^49^ and inhibition of this pathway suppresses the expression of adipogenic transcription factors and further inhibits adipogenesis.^12^ In the present experiment, oil red O staining confirmed that FIL-ADSCs possess a stronger adipogenic ability than CON-ADSCs, but this ability decreased after PIK3CA was knocked down. These results suggest that PIK3CA mutations are closely associated with excessive lipid accumulation in FIL. In addition to hyperplastic fat, underlying skeletal deformities are common in FIL. This may be associated with bone marrow mesenchymal stem cells,^50^ which is a promising direction for further exploration.

The efficacy of surgery for FIL is limited due to the complexity of the surgical procedure, the risk of facial nerve injury, and high recurrence rates.^7^ In recent years, PI3Ka-specific inhibitors have shown promising effects in patients with overgrowth disorders,^13^ although not all patients are sensitive to treatment. One of the potential signaling mechanisms underlying resistance to PI3Kα inhibitors is AKT-independent mTOR activation, which can be realized by PDK1-SGK1 signaling axis activation,^51^ PIM kinase hyperexpression,^52^ and INPP4B-SGK3 activation.^53^ The presence of residual mTOR activity may diminish the effect of PI3Kα inhibitors.^54^ Therefore, dual PI3K/mTOR inhibitors appear to enhance therapeutic efficacy and reduce the likelihood of drug resistance.^55^ We applied the dual-target PI3K-mTOR inhibitor WX390 to FIL-ADSCs and found that it significantly inhibited the overactivation of key signaling molecules in the PI3K pathway. Furthermore, WX390 demonstrated strong potential for inhibiting adipogenesis. We applied WX390 in a mouse model and found that all mice tolerated it well, with significantly inhibited tumor growth and fat formation. These results suggest that WX390 can be used as a therapeutic agent to prevent excessive adipogenesis in FIL. The latest PI3Kα inhibitors can selectively inhibit mutant p110α while sparing wild-type p110α, thereby avoiding common side effects such as hyperglycaemia and hyperinsulinaemia.^56^ However, such drugs may be limited by the varying genotypes in FIL, and their application still needs to be further explored.

To gain further insight into the mechanism underlying the increased adipogenesis caused by PIK3CA-activating mutations, we utilized RNA-seq to identify the mediators linking PIK3CA mutations to excessive lipogenesis in FIL-ADSCs. We observed a negative correlation between PI3K signaling pathway activation and TRPV1 mRNA content. TRPV1 is a member of the transient receptor potential superfamily, and as a calcium channel, it is involved in the response to pain and noxious stimuli.^57^ However, other studies have found that it also inhibits lipid synthesis,^31^ suggesting that it could be a potential target to combat obesity.^37^^,^^58^ These findings have piqued our interest in exploring the role of TRPV1 in regulating adipogenesis in FIL-ADSCs. We discovered that PIK3CA-activating mutations suppressed TRPV1 expression, and overexpression of TRPV1 significantly inhibited the adipogenic ability of FIL-ADSCs. Moreover, we found that capsaicin, an agonist of TRPV1,^59^ also reduced the accumulation of lipid droplets in FIL-ADSCs. Therefore, we hypothesized that TPRV1 may act as a mediator of the ability of PIK3CA mutations to regulate excessive lipid synthesis and may represent a potential therapeutic target.

Our study revealed that PIK3CA mutations increased the adipogenic capacity of FIL-ADSCs by downregulating TRPV1 expression, which might be responsible for the development and progression of FIL. In addition, data on adipogenic inhibition by the dual-target PI3K/mTOR inhibitor WX390 provided promising therapeutic strategies beyond surgery.

### Limitations of the study

In this study, we found that PIK3CA mutations promoted adipogenesis in ADSCs by suppressing TRPV1 expression. Given that TRPV1 is a calcium channel, its overexpression should theoretically result in an elevated intracellular calcium level, but this was not measured. Future research should focus more on the role of TRPV1 in adipogenesis. This includes evaluating the expression of TRPV1 during different stages of adipogenic differentiation, understanding how TRPV1 regulates adipogenic transcription factors, and ascertaining the impacts on lipid metabolism.

## STAR★Methods

### Key resources table

**Table:** 

REAGENT or RESOURCE	SOURCE	IDENTIFIER
**Antibodies**		

Anti-GAPDH antibody	Proteintech	Cat# 60004-1-Ig; RRID:AB_2107436
Anti-α-tubulin antibody	Proteintech	Cat# 11224-1-AP; RRID:AB_2210206
Anti-PIK3CA antibody	Cell Signaling Technology	Cat# 4249; RRID:AB_2165248
Anti-Phospho-Akt (Ser473) antibody	Cell Signaling Technology	Cat# 4060; RRID:AB_2315049
Anti-Akt antibody	Cell Signaling Technology	Cat# 4691; RRID:AB_915783
Anti-Phospho-mTOR (Ser2448) antibody	Cell Signaling Technology	Cat# 5536; RRID:AB_10691552
Anti-mTOR antibody	Cell Signaling Technology	Cat# 2983; RRID:AB_2105622
Anti-PPAR γ antibody	Cell Signaling Technology	Cat# 2435; RRID:AB_2166051
Anti-CD45 antibody	Abcam	Cat# ab40763; RRID:AB_726545
Anti-HLA-DR antibody	Abcam	Cat# ab92511; RRID:AB_10563656
Anti-CD73 antibody	Abcam	Cat# ab313339 (RRID cannot find)
Anti-CD90 antibody	Abcam	Cat# ab3077365 (RRID cannot find)
Anti-CD105 antibody	Abcam	Cat# ab221675 (RRID cannot find)
anti-rabbit IgG	Abcam	Cat# ab270144 (RRID cannot find)

**Biological samples**		

Human facial infiltrating lipomatosis tissue	Shanghai Ninth People’s Hospital	N/A
Human normal facial adipose tissue	Shanghai Ninth People’s Hospital	N/A

**Chemicals, peptides, and recombinant proteins**		

DMSO	MilliporeSigma	Cat# D2650
WX390	Shanghai Jiatan Pharmatech Co., LTD	N/A
Trizol	Takara	Cat# 9108
Collagenase I	Worthington	Cat# LS004196
Matrigel	Corning	Cat# 354234

**Critical commercial assays**		

Cell Counting Kit-8	Epizyme	Cat# CX001M
Modified Oil Red O Staining Kit	Beyotime	Cat# C0158S
PrimeScript™ RT Master Mix	Takara	Cat# RR036A
TB Green Premix Ex Taq	Takara	Cat# RR820A
BCA protein assay kit	Epizyme	Cat# ZJ102
Femto light chemiluminescence kit	Epizyme	Cat# SQ201
RNeasy mini kit	Qiagen	N/A

**Deposited data**		

Raw RNA-sequenced data	This paper	GEO: GSE253170

**Experimental models: Organisms/strains**		

BALB/C nude mice	Shanghai SLAC Laboratory Animal Company	N/A

**Oligonucleotides**		

Primer sequences for RT-qPCR	This paper	Table S2
shRNA targeting sequence for PIK3CA, TRPV1	This paper	Table S3

**Software and algorithms**		

GraphPad Prism 8	GraphPad	N/A
SPSS Statistics version 26.0	IBM	N/A
Image J 1.52v	ImageJ	N/A
R software (4.1.2)	R	N/A
Image-Pro Plus 6.0	Media cybernetics	N/A

### Resource availability

#### Lead contact

The relevant experimental reagents, experimental methods, and related data of this study can be obtained by contacting Xiaoxi Lin (xiaoxilin@shsmu.edu.cn).

#### Materials availability

The study did not generate new unique materials.

#### Data and code availability

The raw sequencing data for this study was uploaded into the GEO database. These data can be obtained at GEO database: GSE253170.

This paper does not report original code.

Any additional information required to reanalyze the data reported in this paper is available from the lead contact upon request.

### Experimental model and study participant details

#### Ethical approval

The “Treatment and mechanism of PI3K/mTOR dual-target inhibitor (WX390) on PIK3CA-related overgrowth spectrum” was established according to the ethical guidelines of the Declaration of Helsinki and was approved by the Ethics Committee of Shanghai Ninth People’s Hospital. The approval number was No.SH9H-2022-T215-1. ADSCs were isolated and collected from adipose tissue, with written consent of donors.

#### Mice

All procedures involving animals were authorized by the Shanghai Ninth People’s Hospital Animal Experimentation Ethics Committee. A total of 1×10^6^ cells (per implant), which were FIL-ADSCs or CON-ADSCs, were suspended in 50 μL DPBS and mixed thoroughly with 50 μL Matrigel (Corning). Both FIL-ADSCs and CON-ADSCs underwent one day of adipogenic induction before being mixed. The cell/Matrigel mixture (100 μL per implant) was injected subcutaneously into the left and right cheeks of 6-week-old female BALB/C nude mice obtained from Shanghai SLAC Laboratory Animal Company. The mice were housed in individual cages with a 12-h light/dark cycle and provided with standard food and water. After 4 weeks, the mice were sacrificed by cervical dislocation, and the implants were harvested and carefully separated from the surrounding tissue. Implants were randomly selected and used for further measurement and staining. Tumor volume (mm^3^) = (shortest diameter)^2^ × (longest diameter) × 0.5.

### Method details

#### ADSC isolation and culture

Facial adipose tissue was obtained from three FIL patients and three patients with other skin lesions (Control) who underwent debulking surgery. The age of these six patients ranged from 3 to 6 years (Table S1). The samples were not subjected to any medical intervention prior to collection. The harvested samples were placed on ice before being transported to the laboratory. The collected adipose tissue was washed with phosphate-buffered saline (PBS) and cut to 0.1-0.2 cm in size. Then, the fat particles were mixed with an equal volume of serum-free solution (Cyagen, USA, HUXMD-90062). The solution contains 0.2% collagenase I (Worthington, USA, LS004196). The mixed solution was incubated in a shaker for 1 h (150 rpm, 37°C). Then, the samples were centrifuged for 5 min at 1000 rpm, and the supernatant and fat suspension were removed. Thirty milliliters of red blood cell lysis buffer (Solarbio, CHN, R1010) was added to the centrifuge tube and centrifuged for 5 min at 1000 rpm.

The supernatant was removed, and ten milliliters of basal medium (Cyagen, USA, HUXMD-90011) was added to the centrifuge tube and pipetted evenly. The cell suspension was filtered to a 10 cm dish using a 100 μm filter and cultured with basal medium in an incubator containing 5% CO_2_ at 37°C. Cells at passages 3-5 were used in the experiments.

#### Characterization of ADSCs

The basal medium was changed every 2 days. When the cells reached 80% confluence, both primary FIL-ADSCs and control (ADSCs isolated from normal facial adipose tissue in facial skin lesions) were washed with PBS for two times and digested with two milliliters of trypsin (Gibco, USA, 25200072) for 2 minutes. Then, two milliliters of basal medium were added to stop digestion. The cell suspension was centrifuged for 5 min at 1000 rpm and the supernatant was removed. After 10^5^ cells were added to 100 μl of flow cytometry staining buffer, surface staining antibodies against CD45, HLA-DR, CD73, CD105, and CD90 (Abcam, UK, EP322Y, EPR3692, EPR28213-52, EPR21846, EPR28145-53) were added for 15 min at room temperature, and the iso control antibody was prepared for staining. Excess antibody was washed off by centrifugation, and flow cytometry staining buffer was added, followed by flow cytometric analysis.

#### DNA extraction and next-generation sequencing

We performed targeted panel sequencing in all cell types using a high-depth NGS approach. The DNA was extracted from cells using the Qiagen DNA Extraction Kit (Qiagen, #13323). Genomic DNA fragments were spliced and modified for sequencing with the NEBNextillinautraII DNA Library Preparation Kit. After library establishment was completed, high-throughput sequencing was performed using the Illumina Nova Seq 6000 platform. The NGS panel had an average sequencing depth of 10,000X and 98% coverage. DNA sequences from the assay samples were compared to the reference sequence hg19 (GRCh37) and analysed to determine the possible mutations.

#### Adipogenic differentiation of ADSCs

Differentiation induction in ADSCs was performed according to the company's instructions. Briefly, both FIL-ADSCs and control ADSCs were placed in 6-well plates at a density of 2x10^5^ per well. When the cells reached 90% confluence, the basal medium was replaced with adipogenic differentiation solution A (Cyagen, USA, HUXMD-90031) for 3 days. Then, solution A was replaced with solution B (Cyagen, USA, HUXMD-90031) for 1 day. The accumulation of lipid droplets was observed by Oil Red O staining after 3 rounds (12 days) of alternating incubations with the A and B solutions.

#### Oil Red O staining

Oil Red O staining was performed according to the instructions of the Modified Oil Red O Staining Kit (Beyotime, C0158S). Quantitative analysis of Oil red O staining area was determined using Image-Pro Plus 6.0 software.

#### RNA extraction and quantitative real-time polymerase chain reaction (RT‒qPCR)

Total RNA was extracted from FIL-ADSCs and control ADSCs using Trizol reagent (Takara, Japan, 9108). cDNA synthesis was performed by following the instructions of PrimeScript™ RT Master Mix (Takara, Japan, RR036A). TB Green Premix Ex Taq (Takara, Japan, RR820A) was used to perform RT‒qPCR assays in an ABI 7900HT system. Glyceraldehyde 3-phosphate dehydrogenase (GAPDH) was used as an internal control. The primer sequences used are listed in Table S2. Relative gene expression was evaluated using the 2^−ΔΔct^ approach.

#### Western blot assay

Total protein from ADSCs was prepared with RIPA lysis buffer (Epizyme, China, PC101) containing 1% phosphatase inhibitor (Epizyme, China, GRF101) and protease inhibitor (Epizyme, China, GRF102) cocktail. Protein concentrations were determined with a BCA protein assay kit (Epizyme, China, ZJ102). The proteins were mixed with protein sample loading buffer (Epizyme, China, LT101) at a 4:1 ratio and then treated at 100°C for 7 minutes before being transferred to 0.45 μm polyvinylidene difluoride (PVDF) membranes. The membranes were placed and blocked in 5% skim milk solution for 2 h at room temperature and then washed with PBST. The membranes were incubated with the corresponding primary antibodies against GAPDH (Proteintech, USA, 60004-1-Ig), α-tubulin (Proteintech, USA, 11224-1-AP), PIK3CA (CST, USA, 4249T), p-AKT-Ser473 (CST, USA, 4060T), AKT (CST, USA, 4691S), p-mTOR (CST, USA, 5536T), mTOR (CST, USA, 2983T), TRPV1 (Proteintech, 66983-1-Ig), PPAR γ (CST, 2435S), FABP 4 (Proteintech, 67167-1-Ig) and C/EBP α (Proteintech, 18311-1-AP) at 4°C overnight. Then, the membranes were washed with PBST and incubated with following horseradish peroxidase (HRP)-conjugated secondary antibody: anti-rabbit IgG (CST, USA, 7074S) or anti-mouse IgG (Proteinech, USA, 66031-1-Ig) at room temperature for 45 minutes. A Femto light chemiluminescence kit (Epizyme, China, SQ201) was used to expose the strips. The expression of the target proteins was determined with Image J software.

#### Cell Counting Kit-8 assay

Cell viability was determined using Cell Counting Kit-8 (Epizyme, China, CX001M) according to the manufacturer’s instructions. Briefly, Cells were seeded into 96-well plates at a density of 3×10^3^ cells per well and cultured with basal medium. At the indicated time point, 10 μL of CCK-8 was added to each well and incubated for 3 hours. The absorbance of each sample, which was proportional to the activity level of cell proliferation, was measured at a wavelength of 450 nm.

#### Histopathological staining

The samples were fixed in 10% (v/v) formalin, followed by dehydration in a series of gradient solutions. The fixed samples were then dehydrated in a gradient alcohol series. The tissue was washed in xylene and embedded in paraffin. The samples were sliced into sections approximately 5 μm thick and stained with haematoxylin and eosin (H&E).

#### Immunohistochemical staining

For immunohistochemistry, tissue sections were placed in a retrieval box filled with citrate buffer (pH 6.0) for antigen retrieval. The slides were washed in PBS on a decolorizing shaker. The sections were then incubated in a 3% hydrogen peroxide solution in the dark at room temperature for 25 minutes, followed by washing with PBS. Blocking was performed with 3% BSA for 30 minutes. The sections were then incubated with the p-AKT-Ser473 or TRPV1 antibody at 4°C overnight, followed by coincubation with the HRP-conjugated anti-rabbit IgG (Abcam, ab270144) for 1 h. To quantify protein expression, the average optical density (OD) value of positive staining was determined by the Image-Pro Plus 6.0 software.

#### Immunofluorescent staining of tissue sections

Sections of tissue were first deparaffinized, followed by antigen retrieval using either EDTA (pH=9.0) or sodium citrate (pH=6.0) solutions, applying high pressure for 2 minutes. Afterward, the sections were incubated with 10% goat serum at ambient temperature for one hour to block non-specific binding, then treated with primary antibodies overnight at 4°C. The sections underwent three washes with Phosphate Buffered Saline before being incubated with Alexa Fluor 488 goat anti-rabbit (A-11008, Invitrogen) and Alexa Fluor 594 goat anti-mouse (ab150116, Abcam) secondary antibodies for 40 minutes at room temperature. Finally, sections were coverslipped using a 4′,6-diamidino-2-phenylindole (DAPI) based mounting medium (ZLI-9557, ZSGB-BIO). The primary antibodies used for immunofluorescent staining included: CD73 (Abcam, UK, EPR28213-52), and CD90 (Proteintech, USA, EPR28145-53).

#### Lentivirus transfection

The lentiviral vector containing short hairpin (sh) RNAs against human PIK3CA and the TRPV1 overexpression lentivirus were constructed by Zuorun Biotechnology (Shanghai, China, Table S3). FIL-ADSCs were placed into 6-well plates (2x10^5^ per well) and cultured until the cell density reached 50%. Then, FIL-ADSCs were infected with virus (MOI=10) for 10 h with 10 μl of polybrene. Afterwards, the virus-containing medium was replaced with basal medium, and incubation was continued for 48 h. Puromycin was used to remove untransfected cells. The expression of PIK3CA and TRPV1 was analyzed by western blotting and RT‒qPCR.

#### RNA sequencing analysis

Total RNA from FIL-ADSCs and FIL-ADSCs treated with WX390 (50 nM, 24 h) was isolated using a RNeasy mini kit (Qiagen, Germany). The establishment of paired-end libraries was performed according to the instructions of the Stranded mRNA-seq Lib Prep Kit for Illumina (ABclonal, China). Differential expression assessment of mRNAs was performed using the R package edgeR. Differentially expressed RNAs with |log2(FC)| values >1, q values <0.05 and a mean FPKM >1 were defined as differentially regulated for subsequent studies. Gene Ontology (GO) evaluation and KEGG pathway analysis (Kyoto Encyclopedia of Genes and Genomes, http://www.genome.ad.jp/kegg) were implemented using the enrich R package (version 3.4.3).

### Quantification and statistical analysis

Data are expressed as the means ± SDs. All assays were repeated more than three times. The statistical significance of differences was evaluated with one-way analysis of variance (ANOVA) or Student's t test. P values < 0.05 were considered significant. All dates are processed by SPSS 26.0 (IBM, USA) and GraphPad Prism software (version 8).
