# Supplementary material for: PIK3CA mutations enhance the adipogenesis of ADSCs in facial infiltrating lipomatosis through TRPV1
Source: iScience. 2024 Jul 5;27(8):110467. doi: 10.1016/j.isci.2024.110467 (PMC11298645; doi:10.1016/j.isci.2024.110467)
Supplement: Document S1. Figures S1 and S2, Tables S1–S3, and Data S1 [file mmc1.pdf]

iScience, Volume *27*

Supplemental information

PIK3CA mutations enhance the adipogenesis of ADSCs in facial infiltrating lipomatosis through TRPV1

Hongrui Chen, Bin Sun, Wei Gao, Yajing Qiu, Wei Wei, Yongguo Li, Wei Ye, Haoliang Song, Chen Hua, and Xiaoxi Lin

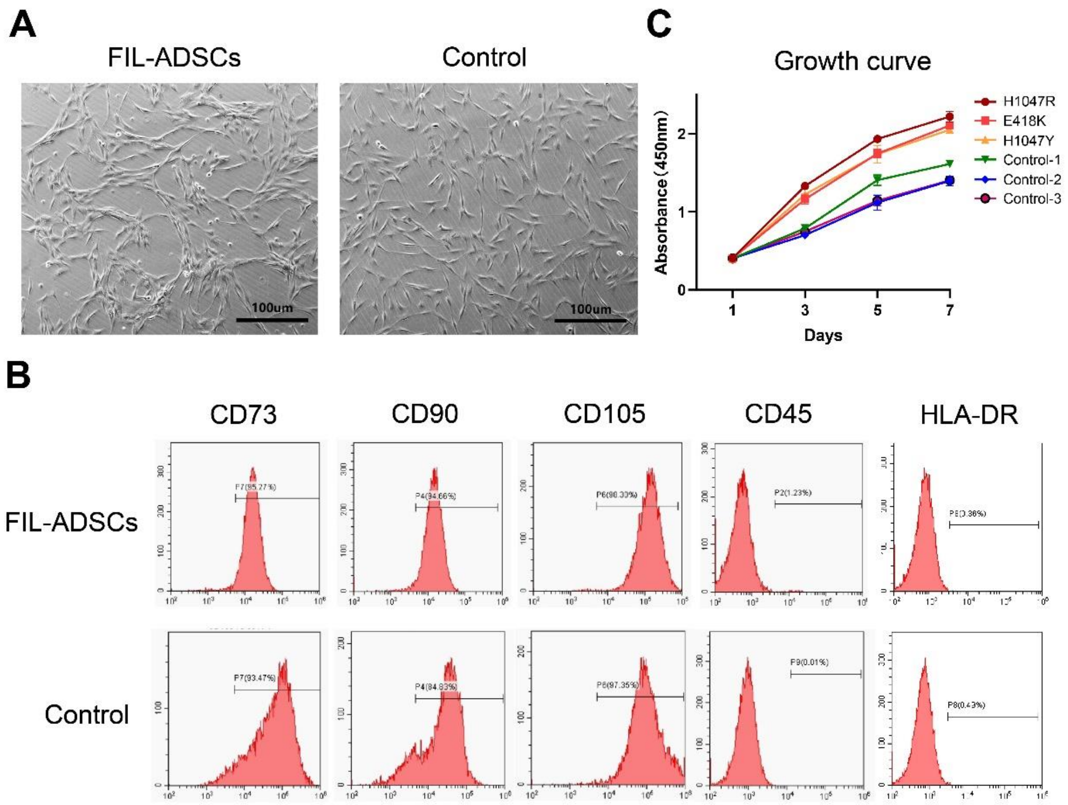

Figure S1: Isolation and characterization of primary adipose-derived mesenchymal stem cells (ADSCs). Related to Figure 2. (A) Morphology of primary ADSCs isolated from FIL patients and controls. (B) The expression of the mesenchymal stem cell surface markers CD73, CD90, and CD105, the haematopoietic marker CD45 and the fibroblastic marker HLA-DR in isolated ADSCs at passage 1 was detected by flow cytometry. (C) Growth curve of six strains of ADSCs. Scale bar: 100 μm.

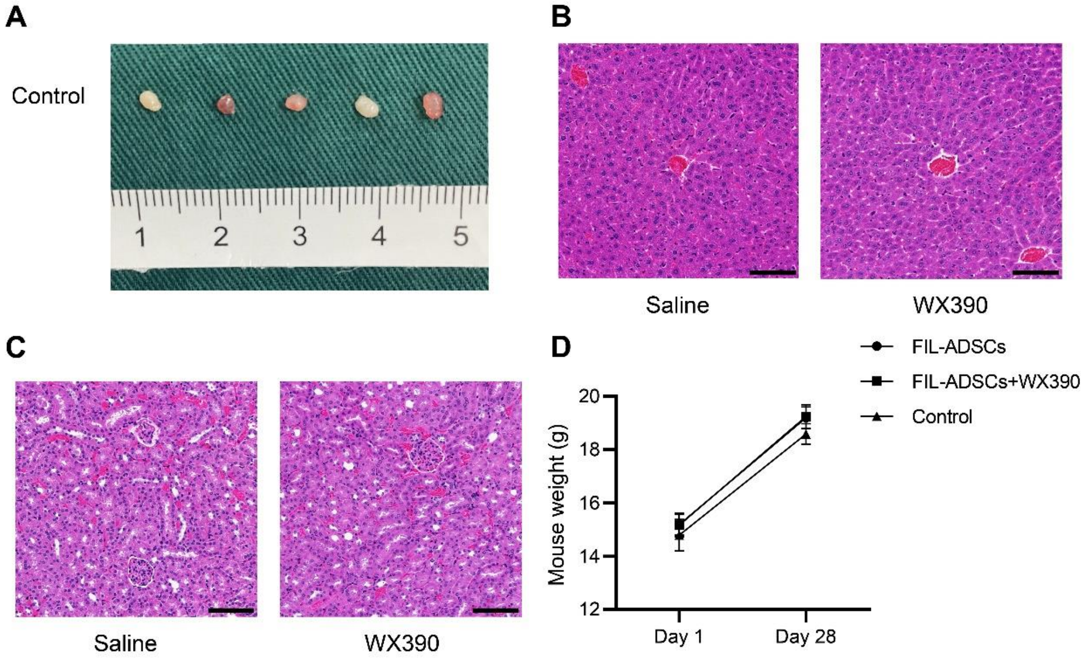

Figure S2: WX390 had no impact on liver and kidney morphology or body weight in mice. Related to Figure 6. (A) Macroscopic appearance of Matrigel implants in the control group. (B) H&E staining of livers from WX390 or saline-treated mice. (C) H&E staining of kidneys from WX390- or saline-treated mice. (D) Body weight of mice that received WX390 and the control treatment during the 4 weeks of treatment. Scale bar: 100 μm.

Patient No.	Sex	Age	Side	Diagnosis	Specimen	Group
1	Male	3	Left	Facial infiltrating lipomatosis	Adipose tissue of lipomatosis	FIL
2	Male	5	Right	Facial infiltrating lipomatosis	Adipose tissue of lipomatosis	FIL
3	Female	6	Right	Facial infiltrating lipomatosis	Adipose tissue of lipomatosis	FIL
4	Female	4	Right	Melanocytic nevus	Normal adipose tissue beyond the margins of the lesion	Control
5	Male	5	Right	Congenital hemangioma	Normal adipose tissue beyond the margins of the lesion	Control
6	Male	5	Right	Congenital hemangioma	Normal adipose tissue beyond the margins of the lesion	Control

Table S1: Clinical information of patients with FIL and patients in control group. Relate to Figure 2. Adipose derived mesenchymal stem cells were extracted from the facial adipose tissue of six patients, including three with facial infiltrating lipomatosis and three with dermatological lesions.

Gene		Primer sequence(5′–3′)	Primer information
H-GAPDH	sense	CATCATCCCTGCCTCTACTGG	NM_001256799.2
	anti-sense	GTGGGTGTCGCTGTTGAAGTC	
H-PPARγ	sense	CTCCAGCATTTCTACTCCACAT	NM_138711.3
	anti-sense	TCCACAGACACGACATTCAA	
H-FABP4	sense	CGAAGTCACTGCAGATGACAG	NM_001442
	anti-sense	TATCCCACAGAATGTTGTAGAGTTC	
H-C/EBPα	sense	GCCTTGTCATTTTATTTGGAGG	NM_004364
	anti-sense	GTTTCCACCCCTTTCTAAGGAC	
H-PIK3CA	sense	TGAGGCTACATTAATAACCATAAAG	NM_006218
	anti-sense	GTCGTCTTGTTTCATCAAAAAATTC	
H-TRPV1	sense	AGATTCGTCAAGCGCATCTTC	NM_018727.5
	anti-sense	CCACAAACAGGGTCTTCATCG	

Table S2: Primers used for quantitative PCR. Related to STAR Methods.

Genes	Primer information
sh-Control	5’-CCTAAGGTTAAGTCGCCCTCG-3’
sh-PIK3CA-1	5’-GCTTGAAGAGTGTCGAATTAT-3’
sh-PIK3CA-2	5’-AGAATATCAGGGCAAGTATAT-3’
Lv-TRPV1	NM_080704
Lv-PIK3CA	NM_006218.4

Table S3: Prime sequence for shRNA. Relate to Figure 4.

Data S1: Original Western blot bands.

AKT

→50kDa

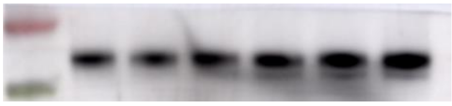

p-AKT

→70kDa
→50kDa

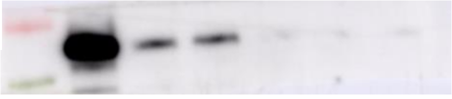

mTOR

→300kDa
→250kDa
→180kDa

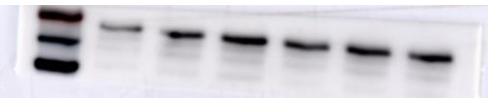

p-mTOR

→300kDa
→250kDa
→180kDa

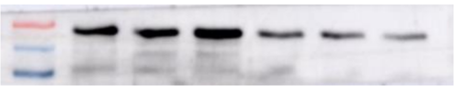

GAPDH

→40kDa

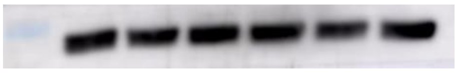

Original Western blot bands Related to Figure 3A

PPAR γ

→55kDa
→42kDa

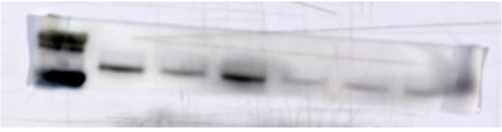

CEBP α

→42kDa
→35kDa
→25kDa

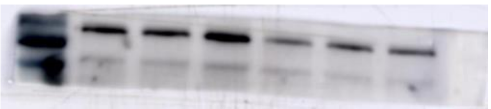

FABP 4

→14kDa

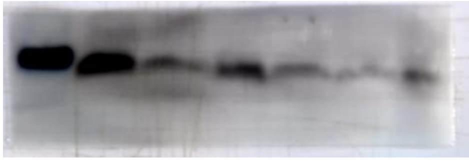

α-tubulin

→55kDa
→42kDa

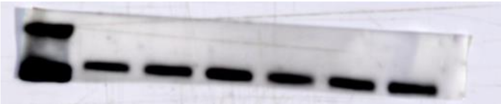

Original Western blot bands Related to Figure 3F

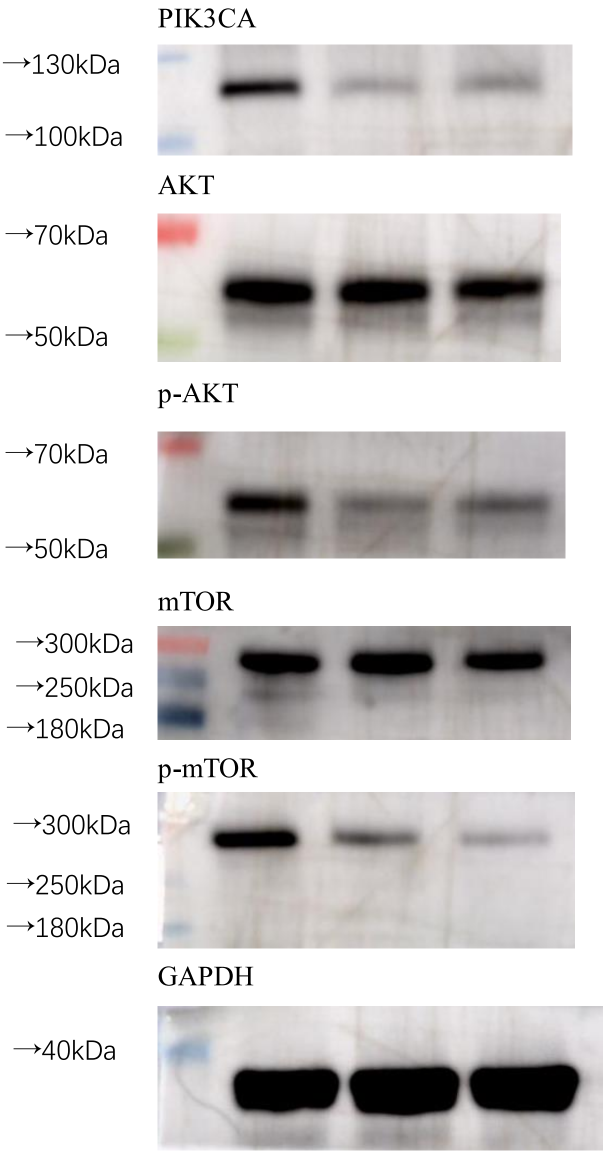

Original Western blot bands Related to Figure 4B

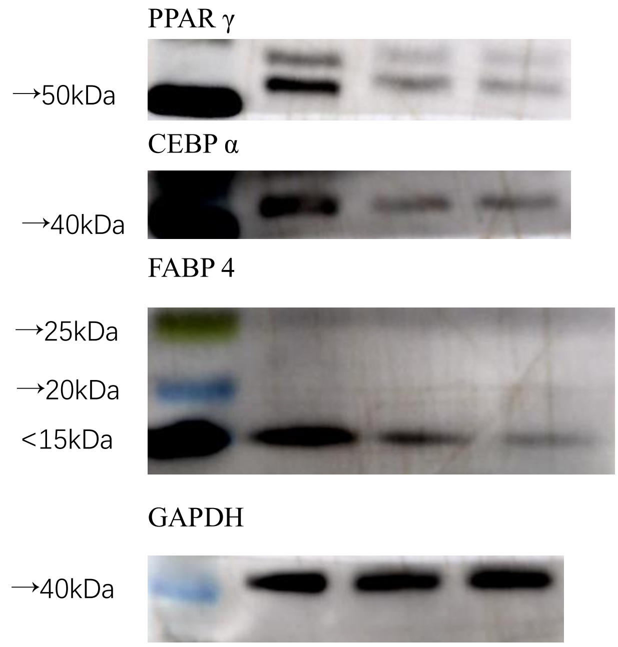

Original Western blot bands Related to Figure 4F

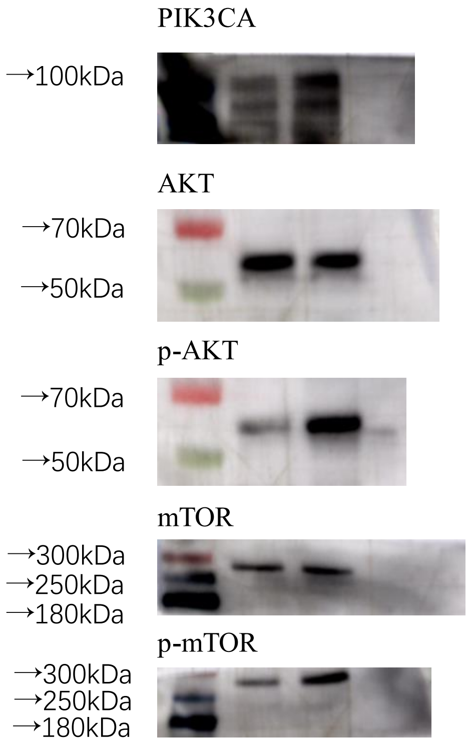

Original Western blot bands Related to Figure 4I

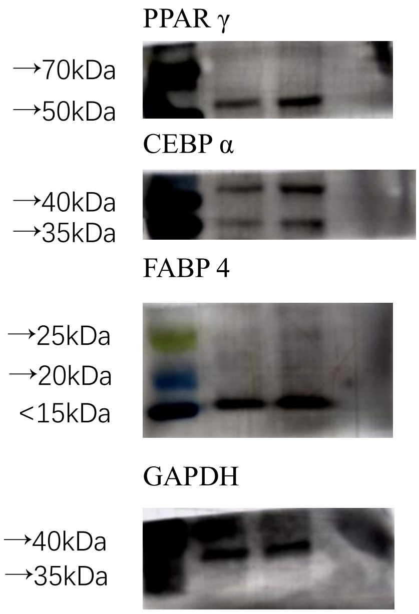

Original Western blot bands Related to Figure 4M

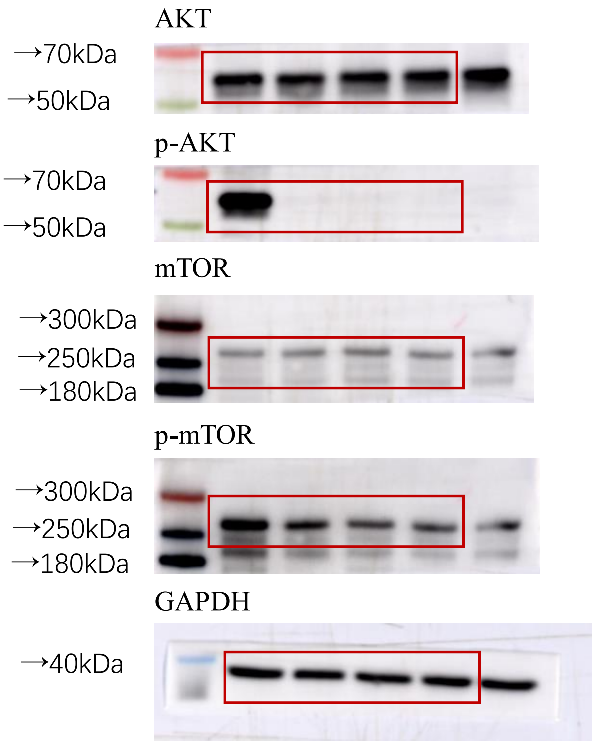

Original Western blot bands Related to Figure 5A

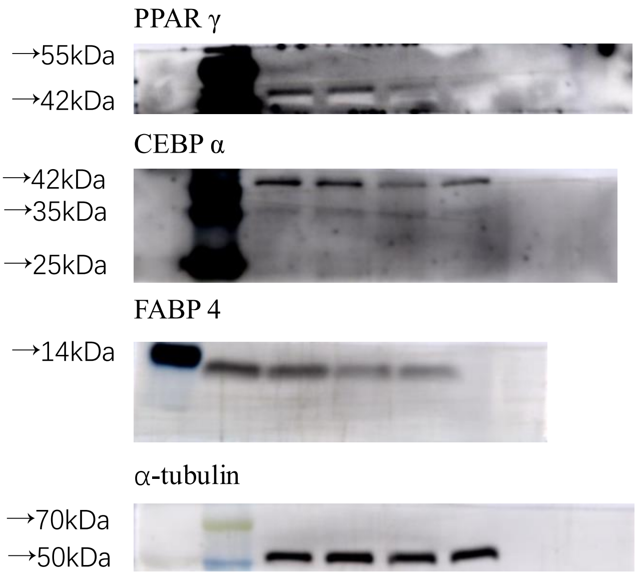

Original Western blot bands Related to Figure 5E

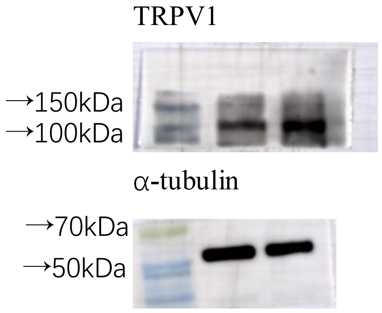

Original Western blot bands Related to Figure 7H

Figure 7J

TRPV1

→130kDa
→100kDa
→70kDa

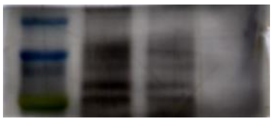

GAPDH

→40kDa
→35kDa

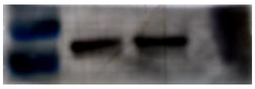

Original Western blot bands Related to Figure 7J

TRPV1

→250kDa
→150kDa
→100kDa

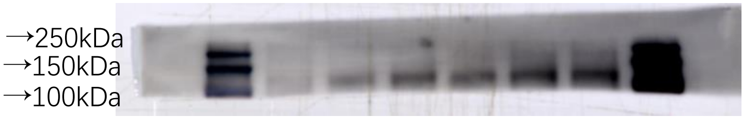

α-tubulin

→55kDa
→42kDa

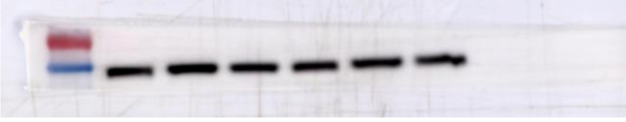

Original Western blot bands Related to Figure 7K

TRPV1

→100kDa

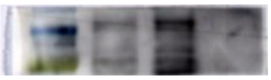

GAPDH

→50kDa
→40kDa
→35kDa

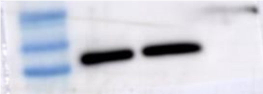

Original Western blot bands Related to Figure 8A

PPAR γ

→100kDa
→70kDa
→50kDa

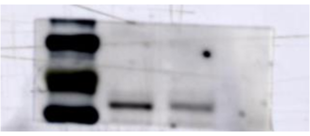

CEBP α

→40kDa
→35kDa

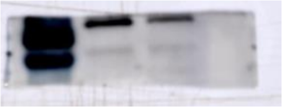

FABP 4

→15kDa

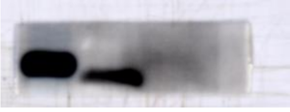

α-tubulin

→50kDa

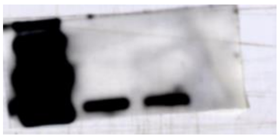

Original Western blot bands Related to Figure 8E

PPAR γ

→70kDa
→50kDa

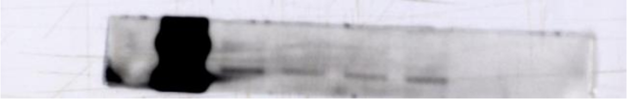

CEBP α

→40kDa
→35kDa
→25kDa

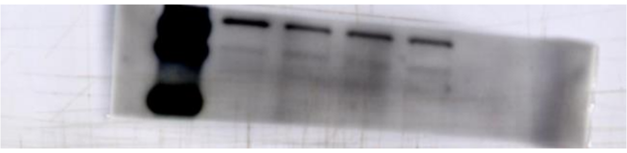

FABP 4

→15kDa

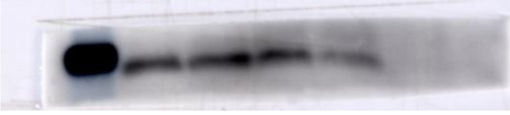

α-tubulin

→70kDa
→50kDa

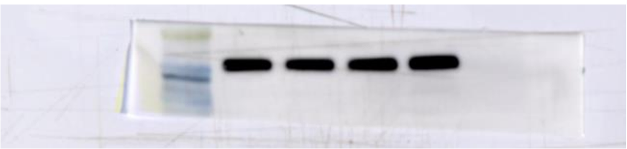

Original Western blot bands Related to Figure 8H
